# Right-Censored Time Series Modeling by Modified Semi-Parametric A-Spline Estimator

**DOI:** 10.3390/e23121586

**Published:** 2021-11-27

**Authors:** Dursun Aydın, Syed Ejaz Ahmed, Ersin Yılmaz

**Affiliations:** 1 Department of Statistics, Faculty of Science, Mugla Sitki Kocman University, Kotekli 48000, Turkey; duaydin@mu.edu.tr; 2 Department of Mathematics and Statistics, Faculty of Science, Brock University, 1812 Sir Isaac Brock Way, St. Catharines, ON L2S 3A1, Canada; sahmed@brocku.ca

**Keywords:** adaptive splines, B-splines, right-censored data, semiparametric regression, synthetic data transformation, time series

## Abstract

This paper focuses on the adaptive spline (A-spline) fitting of the semiparametric regression model to time series data with right-censored observations. Typically, there are two main problems that need to be solved in such a case: dealing with censored data and obtaining a proper A-spline estimator for the components of the semiparametric model. The first problem is traditionally solved by the synthetic data approach based on the Kaplan–Meier estimator. In practice, although the synthetic data technique is one of the most widely used solutions for right-censored observations, the transformed data’s structure is distorted, especially for heavily censored datasets, due to the nature of the approach. In this paper, we introduced a modified semiparametric estimator based on the A-spline approach to overcome data irregularity with minimum information loss and to resolve the second problem described above. In addition, the semiparametric B-spline estimator was used as a benchmark method to gauge the success of the A-spline estimator. To this end, a detailed Monte Carlo simulation study and a real data sample were carried out to evaluate the performance of the proposed estimator and to make a practical comparison.

## 1. Introduction

Time series datasets are censored from the right under specific conditions, such as a detection limit or an insufficient observation process. Consider a device which cannot measure values above a certain point, which is known as a detection limit. Since the device cannot determine the real value of an observation above its detection limit, such observations are recorded as right-censored data points. The hourly observed cloud ceiling heights data collected by the National Center for Atmospheric Research (NCAR) and modelled by [1,2] can be used as an example of a right-censored time series. Although right-censored time series are encountered frequently in the real world, in the literature, there are truly few studies completed on the estimation of right-censored time series. This may be because censorship is an unwanted data irregularity for the researchers, and it is therefore often ignored or solved by outdated techniques.

To solve the censorship problem before modelling the time series, reference [1] used the Gaussian imputation technique to estimate the series using modified ARMA models. In a similar manner, references [2,3] solved the censorship problem by using data imputation techniques. The common ground of these studies is the use of imputation and data augmentation methods to estimate the regression models with autoregressive errors for right-censored time series. On the other hand, there is an easier way to handle the censorship problem called synthetic data transformation. Although data imputation techniques have some merits, they are generally based on iterative algorithms and their calculations are costly. Reference [4] estimated the temporally correlated and right-censored series by Nadaraya–Watson estimator nonparametrically, solving the censorship problem using a data transformation technique. Various data transformation (or synthetic data) methods have been proposed and studied in the literature for independent and identically distributed (i.i.d.) datasets; for example, see [5,6,7]. Because synthetic data transformation manipulates the data structure, which is disadvantageous, this solution method is no longer the preferred technique for right-censored time series. This paper aims to propose a method which can overcome the disadvantage of the synthetic data transformation method.

Note that the studies mentioned above consider the modeling of time series data using parametric or nonparametric methods. The data structure of a time series in the real world is generally not suitable for parametric modelling, because it requires rigid assumptions to reach reasonable estimates. Single-index nonparametric models, on the other hand, are very flexible, which is an important advantage over parametric methods and there are valuable studies on the subject [2,8,9]. However, nonparametric approaches lose their statistical efficiencies, when the number of covariates increases. In addition, it should be noted that, when a time series dataset is right-censored, the weaknesses of both methods are further increased.

Considering the issues mentioned above, this paper adopts semiparametric regression model for estimating right-censored time series. Although several researchers have introduced different types of semiparametric estimators for time series data, such as [10,11], there remains a significant gap in the research regarding the modelling of right-censored time series data. To address this absence, our paper proposes a modified semiparametric A-spline (AS) estimator based on synthetic data transformation. Thus, the bidirectional flexibility of the semiparametric model will be used, and the censorship problem will be effectively solved.

The paper is designed as follows: the methodology and fundamental ideas about right-censored semiparametric time series model with autoregressive errors and the synthetic data transformation method are given in Section 2. Section 3 introduces a modified AS estimator for parametric and nonparametric components of the right-censored time series model, and a semiparametric B-spline (BS) is given as a benchmark. Section 4 involves the statistical properties and evaluation criteria for both the modified AS and benchmark BS methods. Section 5 introduces some additional information about the penalty term of the semiparametric AS approach. Section 6 and Section 7 contain a detailed Monte Carlo simulation study and a real-world data example, respectively. Conclusions are presented in Section 8.

## 2. Background

The classical semiparametric model can be defined as a hybrid model with a finite dimensional parametric component and a nonparametric component having an infinite dimensional nuisance parameter. See [12,13,14,15] for additional information. In both theory and practice, the semiparametric model brings a new perspective to data modeling, since it includes both parametric and nonparametric components. As mentioned in the previous section, it is well-suited to time series data, because it brings the advantages of the semiparametric model to time series analysis.

Suppose that a time series dataset $\left\{Z_{t},\;x_{t},\;s_{t},\;t=1,2,\ldots ,n\;\;\right\}$ satisfies an uncensored semiparametric time series model of the form:

(1) $$Z_{t}=\;x_{t}\beta +f\left(s_{t}\right)+\varepsilon_{t},\;\;a=s_{1}<\ldots <s_{n}=b,$$

where $Z_{t}{}^{\prime }$s are the observations of stationary time series, $x_{t}=\left(x_{t1},\ldots ,x_{tp}\right)$ and $x_{1},\ldots ,x_{n}$ are known *p*-dimensional vectors of the explanatory variables, $\beta ={\left(\beta_{1},\beta_{2},\ldots ,\beta_{p}\right)}^{\prime }$ is an unknown p-dimensional vector of the regression coefficients to be estimated, f(.) is an unknown smooth function that describes the relationship between $Z_{t}$ and a nonparametric temporal covariate $s_{t}$, and finally, $\varepsilon_{t}$’s are the stationary autoregressive error terms generated by:

(2) $$\varepsilon_{t}=\rho_{1}\varepsilon_{t-1}+\ldots +\rho_{k}\varepsilon_{t-k}+u_{t},$$

where $\rho_{1},\ldots ,\rho_{k}$ are the autoregressive coefficients, and $u_{t}$ denotes the independent and identically distributed random error terms with mean zero and a constant variance. Model (1) does not include lagged $Z_{t}{}^{\prime }$s and has auto-correlated errors. This expression makes it a suitable model for the semiparametric regression analysis of certain kinds of time series.

A common problem in practice is that dependent observations $Z_{t}{}^{\prime }s$ cannot be perfectly collected due to limitations including the detection limit of an evaluation tool or the end time for the study. To express this situation algebraically, we assume that $Z_{t}{}^{\prime }s$ are censored from the right by a non-negative random variable representing detection limit $C_{t}$. Therefore, instead of observing the values of $Z_{t}$, we now observe:

(3) $$Y_{t}=\min\left(Z_{t},C_{t}\right)\;\mathrm{and}\;\delta_{t}=\{\begin{array}{c} 1\;\;if\;Z_{t}\leq C_{t}\;\;\left(\mathrm{uncensored}\right)\\ 0\;\;if\;Z_{t}>C_{t}\;\;\left(\mathrm{censored}\right)\;\;\;\; \end{array},$$

where $\delta_{t}$’s denote the censoring information. Suppose that we are interested in estimating the mean semiparametric regression function. The distribution of the observable random variables does not identify the mean regression function uniquely. However, this problem can be solved as follows.

Let $F_{Z}\left(\alpha \right)=P\left(Z\leq \alpha \right),\;\;G_{C}\left(\alpha \right)=P\left(C\leq \alpha \right)$, and $H_{Y}\left(\alpha \right)=P\left(Y\leq \alpha \right)$ for α∈ℝ be cumulative distribution functions of non-negative random variables $Z_{t},\;\;C_{t},\;\mathrm{and}\;Y_{t},$ respectively. If random variables $Z_{t}$ and $C_{t}$ are independent, then the survival function ${\bar{H}}_{Y}\left(\alpha \right)$ for observed response variable $Y_{t}$ can be defined from the basic relationship between $F_{Z}\;\mathrm{and}\;G_{C}$:

(4) $$\left\{{\bar{H}}_{Y}\left(\alpha \right)=1-H_{Y}\left(\alpha \right)\right\}=\left[\left(1-F_{Z}\left(\alpha \right)\right)\cdot \left(1-G_{C}\left(\alpha \right)\right)\right].$$

Given a random sample from the distribution of ($Y_{t},X_{t},s_{t},\;\delta_{t}$), it is of interest to examine the explanatory variables’ effect on the observations of time series (i.e., response variable) by estimating the survival function ${\bar{H}}_{Y}\left(\alpha \right)=P\left(Y>\alpha \right)$, which is the regression function $E\left(Y_{t}|x_{t},s_{t}\right)=x_{t}\beta +f\left(s_{t}\right)$, the conditional mean of time series $Y_{t}$. However, because of the censoring, ordinary methods cannot be applied directly to estimate the regression function. To overcome censored observations, a data transformation technique should be used. One of the most widely used techniques is the synthetic data transformation, detailed in the section below.

### Synthetic Data

To extend the penalized sum of squares approach to right-censored semiparametric regression analysis, we updated the synthetic data approach developed by [5]. The first step is to create an unbiased synthetic response variable of which the expectation is equal to the original and then to obtain the penalized squares estimator by means of this synthetic variable. The main goal of this transaction is to consider the censoring effect on the distribution of response variable. In the case of censored data, the authors of [16,17] used the synthetic data approach.

In the synthetic approach, we replace observed variable $Y_{t}$ with transformed data $Y_{tG}$; a transformation maintains the conditional expectation of original variable $Z_{t}$. To describe this situation, it is easier to proceed directly using the cumulative distributions given in Lemma 1 below. Note also that if $G_{C}$ is known then it is possible to transform observed data $\left\{(Y_{t},\;\delta_{t}),\;t=1,\ldots ,n\right\}$ into unbiased synthetic data, given by:

(5) $$Y_{tG}=\frac{\delta_{t}Y_{t}}{1-G_{C}\left(Y_{t}\right)},$$

where $G_{C}\left(.\right)$ is the distribution function of the censoring time $C_{t}$, as defined before. It should be noted that the distribution of $G_{C}$ is rarely known. In this case, we use the Kaplan–Meier estimator defined by:

(6) $$1-{\hat{G}}_{c}\left(y\right)=\prod_{t=1}^{n}{\left(\frac{n-t}{n-t+1}\right)}^{I\left[Y_{\left(t\right)}\leq y,\;\;\;\delta_{\left(t\right)}=0\right]},\;y\geq 0,$$

where $Y_{\left(1\right)}\leq \ldots \leq Y_{\left(n\right)}$ are the sorted values of $Y_{1},\ldots ,Y_{n}$ and $\delta_{\left(t\right)}$ is the $\delta_{t}$ related to $Y_{\left(t\right)}$. Equation (5) has the following properties: (a) if distribution $G_{C}$ is selected arbitrarily, some $Y_{\left(i\right)}$ can be identical. In this case, the ranking of $Y_{1},\ldots ,Y_{n}$ into $Y_{\left(1\right)}\ldots Y_{\left(n\right)}$ is not unique. However, the Kaplan–Meier estimator allows us to define the ranking of $Y_{t}$ uniquely; (b) ${\hat{G}}_{C}\left(.\right)$ has jumps only at the censored observations of the time series (see [18]).

Substituting ${\hat{G}}_{C}\left(.\right)$ for $G_{C}\left(.\right)$ in Equation (5), we construct the following synthetic data, given by:

(7) $$Y_{t\hat{G}}=\frac{\delta_{t}Y_{t}}{1-{\hat{G}}_{C}\left(Y_{t}\right)}.$$

Then, one practical consequence of the following Lemma is that synthetic data $Y_{t\hat{G}}$ and completely observed response times $Z_{t}$ have the same conditional expectations, as claimed in before.

**Lemma** **1.** *Consider time series data*$Z_{t}$*denoted as a response variable. If the data is censored by random censoring variable*C*with distribution*$G_{C}$*, transform observed series*$Y_{t}=min\left(Z_{t},C_{t}\right)$*to*$Y_{tG}=\delta_{t}Y_{t}/1-G_{C}\left(Y_{t}\right)$*in an unbiased form, as defined in Equation (4). Based on the information, it can be easily verified that*$E\left[Y_{tG}|x_{t},s_{t}\right]=E\left[Z_{t}|x_{t},s_{t}\right]=x_{t}\beta +f\left(s_{t}\right)$*. However, generally,*$G_{C}$*is unknown as mentioned before. Therefore,*$Y_{t\hat{G}}$*is used which is defined in Equation (7), instead of*$Y_{tG}$*. Because of*${\hat{G}}_{c}\to G$*when*n→∞*, (see [5]), it is ensured that*$E\left[Y_{t\hat{G}}|x_{t},s_{t}\right]≅E\left[Y_{tG}|x_{t},s_{t}\right]=x_{t}\beta +f\left(s_{t}\right)$.

Let us consider that $\tau_{H_{Y}}=\sup\left\{\alpha :H_{Y}\left(\alpha \right)<1\right\}$, where $H_{Y}\left(.\right)$ is defined right after Equation (3). In the literature, the convergence rate of the Kaplan–Meier estimator is examined in two classes: (i) restriction of time-interval as [0, α] with $\alpha <\tau_{H_{Y}}$; (ii) extension of time-interval $\left[0,\;\tau_{H_{Y}}\right]$ (see [19] for more detailed discussions). Here, the convergence rate of the Kaplan–Meier estimator is inspected with regard to case (ii). However, $\left[0,\;\tau_{H_{Y}}\right]$ cannot be used without some strong conditions that can be given by:

(i) $G\left(\tau_{H_{Y}}\right)<1=F\left(\tau_{H_{Y}}\right)$;
(ii) $\tau_{H_{Y}}<\infty$;
(iii) $\int_{0}^{\tau_{H_{Y}}}\frac{1}{1-G\left(\alpha \right)}dF<\infty$.

Details about conditions (i)–(iii) were studied by [20]. The convergence of $\hat{G}\to G$ over the interval $\left[0,\;\tau_{H_{Y}}\right]$ can be provided. Reference [19] clearly shows both strong and weak convergences at the rate $n^{-\vartheta }$ where 0≤ϑ≤1/2.

The proof of Lemma 1 is given in Appendix A.

The major concern of this paper is to overcome the censoring problem and to estimate the semiparametric time series model efficiently. To achieve this goal, we used two different approaches, BS and modified AS estimators. In the following section, we applied these approaches to the transformed data to estimate time series observations under random right-censorship.

## 3. Estimating the Semiparametric Model Based on the BS Estimator

We first introduce the BS considered for estimating the components of model (1). A univariate B-spline is constructed by a piecewise polynomial function of degree q such that its derivatives up to order (q−1) is continuous at each knot point $r_{1},\ldots ,\;r_{k}.$ The set of BSs of degree q over the real numbers $\left(r_{1},\ldots ,r_{k}\right)=r$ is a vector space of dimension q+k+1. In addition, note that k denotes the number of interior knots, while q≥0 indicates the polynomial order. For example, the polynomials of order q=0, 1, 2, and 3 are defined as constant, linear, quadratic, and cubic BS basis functions, respectively. If the knots are equally spaced (i.e., separated by same distance $h=(r_{k+1}-r_{k}$)), the knot points and the corresponding BSs are called uniform.

**Definition** **1.** *Given an ordered knot vector* $r=\left\{r_{1}\leq r_{2}\leq \ldots \leq r_{k}\right\}$ *in the domain of covariate* $s_{t}$ *, then* $i^{th}$ *BS basis functions* $\left\{B_{i,q}\left(s_{t}\right),\;\;i=1,\;2,\ldots ,q+k+1\right\}\;$ *of degree* q=0 *and* q>0 *can be defined in recursive series, respectively, as:*

(8) $$\;B_{i,0}\left(s\right)=\{\begin{array}{cc} 1 & if\;\;\;r_{i}\leq s\leq r_{i+1}\\ 0, & \;\;\;\;otherwise \end{array},$$

(9) $$B_{i,q}\left(s\right)=\frac{s-r_{i}}{r_{i+q}-r_{i}}B_{i,q-1}\left(s\right)+\frac{r_{i+q+1}-s}{r_{i+q+1}-r_{i+1}}B_{i+1,q-1}\left(s\right).$$

*Note that if the denominator of Equation (9) is equal to zero, then the BS basis function is assumed to be zero. From Equations (8) and (9), a set of* (q+k+1) *basis functions have the following important properties:*

*(a) The BS basis functions form a partition of unity,* $\overset{q+k+1}{\underset{i=1}{\sum }}B_{i,q}\left(s\right)=1$ *;*

*(b) For all values of covariate*$s_{t},$ $\;B_{i,q}\left(s\right)\geq 0$*; and*

*(c)*$\;B_{i,q}\left(s\right)$*is realized in the interval [*$r_{k},\;r_{k+q+1}$*]*.

Reference [21] proposes an algorithm to solve equation (9). See also the work of [22] for more detailed discussions on the BS approximation. Note also that the BS curve can be uniquely represented as a linear combination of the BSs basis functions in Equation (9), as given in the next section. Note that references [23,24] could be counted as recent studies about BSs.

### 3.1. BS Estimator

As previously noted, in this paper, we fit semiparametric time series model (1) with right-censored data. For this purpose, the BS estimator can be used as an approximation method. Using the synthetic data in Equation (7), we estimated the parametric and nonparametric components of model (1). Therefore, the sum of the squares of the differences between the censored time series values $Y_{t\hat{G}}$ and $\left(x_{t}\beta +f\left(s_{t}\right)\right)$ are minimum. Assume that f(.) is a smooth function that can be approximated by a linear combination of the BSs basis functions in Equations (8) and (9):

(10) $$f\left(s\right)\;≅\;\sum_{i=1}^{m=q+k+1}\alpha_{i}B_{i,q}(s)=B\alpha \;,$$

where m=(q+k+1) is the total number of BS basis functions being used, ${\hat{\alpha }}_{i}{}^{\prime }s$ are estimated coefficients (or control points) for each BS, B is an (n×m)-dimensional matrix which includes BSs as defined by Equation (9) and $\alpha =\left(\alpha_{1},\ldots ,\alpha_{m}\right){}^{\prime }$ is a parameter vector of the BS function. Note also that the autoregressive errors in model (1) follow an n-dimensional multivariate normal distribution with a zero mean and stationary (n×n) covariance matrix Σ, that is, ${\left(\varepsilon_{1},\ldots ,\varepsilon_{n}\right)}^{T}∼N_{n}\left(0,\;\Sigma \;\right),$ where the covariance matrix Σ is a symmetric and positive definite matrix with elements:

(11) $$\Sigma =\frac{\sigma_{u}^{2}}{1-\rho^{2}}R,\;\;R\left(t,j\right)=\rho^{\left|t-j\right|},\;\;1\leq \left(t,j\right)\leq n.$$

Throughout the paper, the notation is used as $\Sigma^{-1}=V$. Note that V is generally unknown. However, its elements can be obtained by the generalized least squares (GLS) based on an iterative process. Then, as in [25] which is a penalized BS study combining BS and difference penalties, the estimates of the components of semiparametric model (1) were obtained by minimizing the penalized sum of squares (PSS) criterion:

(12) $$PSS=\sum_{t=1}^{n}V{\left\{Y_{t\hat{G}}-\sum_{j=1}^{p}x_{tj}\beta_{j}-\sum_{i=1}^{m}\alpha_{i}B_{i,q}(s)\right\}}^{2}+\lambda \sum_{i=q+1}^{m}{\left(\Delta^{q}\alpha_{i}\right)}^{2},$$

where $∆\alpha_{i}=\left(\alpha_{i}-\alpha_{i-1}\right)$ is the first-order difference penalty on the coefficients of the BSs. The other differences can be defined as follows:

(13) $${∆}^{2}\alpha_{i}=∆\left(∆\alpha_{i}\right)=\left(\alpha_{i}-\alpha_{i-1}\right)-\left(\alpha_{i-1}-\alpha_{i-2}\right)=\alpha_{i}-2\alpha_{i-1}+\alpha_{i-2},$$

and similarly:

(14) $$\Delta^{q}\alpha_{i}=∆\left(\Delta^{q-1}\alpha_{i}\right).$$

Note that if degree q=0 in Equation (12), we obtain semiparametric ridge regression based on BSs. When λ=0 in Equation (12), we have the minimization equation of ordinary least squares regression with a correlated error. If λ>0, the penalty only influences the main diagonal and q sub-diagonals (on both sides of the main diagonal elements) of the banded structure system due to the limited overlap of the BSs.

We rewrite the minimization criterion described as Equation (12) in a matrix and vector notation:

(15) $$PSS={\left(Y_{\hat{G}}-X\beta -B\alpha \right)}^{\prime }\;V\;\left(Y_{\hat{G}}-X\beta -B\alpha \right)+\lambda \|D\alpha {\|}^{2},$$

where ‖.‖ denotes Euclidean norm, $X=\left(x_{1},\ldots ,x_{n}\right){}^{\prime }$, $Y_{\hat{G}}=\left(Y_{1\hat{G}},\ldots ,Y_{t\hat{G}}\right){}^{\prime }$ is the synthetic response vector defined in Equation (7), λ>0 is a smoothing parameter, and D denotes the matrix notation of the difference operator $\left({∆}^{q}\right)$ defined in Equation (13). For example, D is an (n−2)×n-dimensional banded matrix that corresponds to the second-order difference penalty, given by:

(16) $$D=\left[\begin{array}{ccc} 1 & -2 & \begin{array}{ccc} 1 & \cdots & 0 \end{array}\\ \vdots & \ddots & \begin{array}{ccc} \ddots & \ddots & \;\vdots \end{array}\\ 0 & \cdots & \begin{array}{ccc} 1 & -2 & 1 \end{array} \end{array}\right].$$

From simple algebraic operations, it follows that the solution to the minimization problem in Equation (15) satisfies the following block matrix equation:

(17) $$\left(\begin{array}{cc} X^{\prime }V\;X & X^{\prime }V\;B\\ B^{\prime }V\;X & \left(B{}^{\prime }V\;B+\lambda D^{\prime }D\right) \end{array}\right)\left(\begin{array}{c} \beta \\ \alpha \end{array}\right)=\left(\begin{array}{c} X^{\prime }\\ B^{\prime } \end{array}\right)VY_{\hat{G}}.$$

Given a parameter λ>0, the corresponding estimators based on BSs for vectors β and α can be easily obtained by:

(18) $${\hat{\alpha }}_{BS}={\left[B^{\prime }VB+\lambda D^{\prime }D\right]}^{-1}B^{\prime }V\left(Y_{\hat{G}}-X{\hat{\beta }}_{BS}\right),$$

and:

(19) $${\hat{\beta }}_{BS}={\left[\left(X^{\prime }V-A_{BS}\right)X\right]}^{-1}\left(X^{\prime }-A_{BS}\right)\;VY_{\hat{G}},$$

where $A_{BS}=X^{\prime }VB{\left[B^{\prime }VB+\lambda D^{\prime }D\right]}^{-1}B^{\prime }V.$ It should be noted that the estimates of the unknown regression function in a censored semiparametric model are obtained by:

(20) $${\hat{f}}_{BS}=B{\hat{\alpha }}_{BS}=\left[\hat{f}\left(s_{1}\right),\ldots ,\hat{f}\left(s_{n}\right)\right]{}^{\prime }.$$

From Equations (19) and (20), we see that the fitted values of dependent time series data can be written as:

(21) $${\hat{\mu }}_{BS}=\left(X{\hat{\beta }}_{BS}+{\hat{f}}_{BS}\right)=H_{BS}Y_{\hat{G}}=E\left[Y\;|\;X,\;s\right],$$

where $H_{BS}$ is a hat matrix for BSs and computed as follows:

(22) $$H_{BS}=[X{\left[\left(X^{\prime }V-A_{BS}\right)X\right]}^{-1}\left(X^{\prime }-A_{BS}\right)V\left(I-M_{BS}\right)+M_{BS}],$$

where $M_{BS}=B{\left[B^{\prime }VB+\lambda D^{\prime }D\right]}^{-1}B^{\prime }V$**.**

### 3.2. AS Estimator

The adaptive spline (AS) applies an adaptive ridge penalty to the BS method, which makes it more flexible for knot determination. The AS concept is explained in [26] in a nonparametric context. However, in this paper, we generalized this estimation concept to the semiparametric environment based on synthetic response observations. It should be noted that the location and number of knots have crucial importance in terms of synthetic data transformation. This issue is discussed in detail in Section 4.3. The point here is that a more efficient estimator based on synthetic responses is needed, as most of the existing smoothing techniques (spline smoothing, kernel smoothing, etc.) cannot properly handle synthetic data. This article aims to solve this issue with the AS estimator.

When a BS is defined on the knots $r_{1}\leq r_{2}\leq \ldots \leq r_{k}$ such that $\Delta^{q}\alpha_{i}=0$ for some $i^{th}$ knot, it may be reparametrized as a BS on the knots $r_{1},r_{2},\ldots ,r_{i-1},r_{i+1},\ldots ,r_{k}$. Accordingly, when m=(q+k+1), we want to put a penalty on the number of non-zero differences indicated as below:

(23) $$\lambda \overset{m}{\underset{i=q+1}{\sum }}{\left\|\Delta^{q}\alpha_{j}\right\|}_{0}\;,$$

where $\Delta^{q}\alpha_{i}$ is the $q^{th}$-order difference operator and ${\left\|\Delta^{q}\alpha_{i}\right\|}_{0}$ is the $L_{0}$-norm of the differences, that is, ${\left\|\Delta^{q}\alpha_{i}\right\|}_{0}$ = 0 if $\Delta^{q}\alpha_{j}=0$, otherwise, ${\left\|\Delta^{q}\alpha_{i}\right\|}_{0}$ = 1, and λ is a positive penalty parameter that ensures the tradeoff between the goodness of fit to the data and the smoothness of the fitted curve. This penalty enables us to remove knot $r_{i}$ that is not related to the smoothing problem, to join the neighbor intervals $\left[r_{i-1},r_{i}\right)$ and $\left[r_{i},\;r_{i+1}\right)$, and to carry on fitting with a BS described over the remaining knot points. Note also that when λ→0, the fitted curve becomes a BS with knots $r_{i},i=1,\;2,\ldots ,k$ and when λ→∞, the fitted function becomes a polynomial of degree q.

It should be emphasized that one of the important points about the adaptive ridge penalty is that Equation (23) cannot be differentiated due to the $L_{0}$-norm. As a result, the fitting process is made numerically untraceable. An approximate solution to dealing with the $L_{0}$-norm is provided by [27,28]. Following the studies of these authors, we approximate the $L_{0}$-norm by using an iterative process referred to as an “adaptive ridge” based on synthetic data. The new criterion function is expressed by the following weighted penalized sum of squares:

(24) $$WPSS={\left(Y_{\hat{G}}-X\beta -B\alpha \right)}^{\prime }V\left(Y_{\hat{G}}-X\beta -B\alpha \right)+\lambda \sum_{i=q+1}^{m}w_{i}{\left({\;\Delta }^{q}\alpha_{i}\right)}^{2},$$

where $w_{i}$’s denote the positive weights. It should be noted that the penalty is close to the $L_{0}$-norm of the differences when the weights are iteratively calculated from the parameter vector α of BS following the equation:

(25) $$w_{i}={\left[{\left(\Delta^{q}\alpha_{i}\right)}^{2}+\gamma^{2}\right]}^{-1},\;\;\gamma >0,$$

where γ is a constant properly determined by the researcher.

**Remark** **1.** *There are a few important points to know about the selection of* γ *. If* $\left(\Delta^{q}\alpha_{i}\right)<\gamma$ *, then the magnitudes of* $w_{i}$ *’s might be quite large, resulting in* $\left(\Delta^{q}\alpha_{i}\right)\;≅\;0$ *and the penalty term turning into* $w_{i}{\left(\Delta^{q}\alpha_{i}\right)}^{2}\;≅\;0$ *. Furthermore, if* $\left(\Delta^{q}\alpha_{i}\right)\gg \gamma$ *, then* $w_{i}{\left(\Delta^{q}\alpha_{i}\right)}^{2}\;≅\;{\left\|\Delta^{q}\alpha_{i}\right\|}_{0}$ *. This convergence gives us a measure of how relevant the* $i^{th}\;$ *knot point is. In practice, one possible choice, suggested by [28], is* $\gamma =10^{-5}$ *. They select the knots (denoted as* $r_{i^{*}}$ *) with a weighted difference bigger than 0.99. The number of parameters of the chosen BS is* $m_{\lambda }=q+k_{\lambda }+1$ *, where* $k_{\lambda }$ *denotes the number of selected knot points.*

Note that reference [28] provides a figure to show the effects of different norm degrees (q) on the quality of estimation. It is seen from that the performance of estimation does not change for different values of γ when norm degree is zero (q=0). However, it affects the performance seriously if q>0.

For some λ>0 and non-negative weights, the WPSS of Equation (26) can be rewritten as:

(26) $$WPSS={\left(Y_{\hat{G}}-X\beta -B\alpha \right)}^{\prime }V\left(Y_{\hat{G}}-X\beta -B\alpha \right)+\lambda \alpha^{\prime }K\alpha ,$$

where K is a penalty matrix and written as $\;K=D{}^{\prime }WD$, where $W=\mathrm{diag}\left(w_{q+1},\ldots ,w_{m}\right)$ and D is the matrix form of the difference operator ${\;\Delta }^{q}$, as defined in Equation (13). Simple algebraic operations show that the solution to the minimization problem WPSS in Equation (26) satisfies the block matrix equation:

(27) $$\left(\begin{array}{cc} X^{\prime }VX & X^{\prime }VB\\ B^{\prime }VX & \left(B^{\prime }VB+\lambda K\right) \end{array}\right)\left(\begin{array}{c} \beta \\ \alpha \end{array}\right)=\left(\begin{array}{c} X^{\prime }\\ B^{\prime } \end{array}\right)VY_{\hat{G}}.$$

By similar arguments as in the case of the BS approach, the corresponding estimators ${\hat{\alpha }}_{AS}$ and ${\hat{\beta }}_{AS}$ of α and β, based on the right-censored semiparametric time series model (1) with correlated data, can be easily obtained, respectively, as:

(28) $${\hat{\alpha }}_{AS}={\left[B^{\prime }VB+\lambda K\right]}^{-1}B^{\prime }V^{\prime }\left(Y_{\hat{G}}-X{\hat{\beta }}_{AS}\right),$$

and:

(29) $${\hat{\beta }}_{AS}=({\left(X^{\prime }V-A_{AS})X\right)}^{-1}\left(X^{\prime }-A_{AS}\right)VY_{\hat{G}},$$

where $A_{AS}=X^{\prime }VB{\left[B^{\prime }VB+\lambda K\right]}^{-1}B^{\prime }V^{\prime }$. The proofs and derivations of Equations (28) and (29) are given in Appendix B. Notice that the estimates corresponding to the nonparametric part of the semiparametric model (1) are obtained using Equation (28) as described in the following equation:

(30) $${\hat{f}}_{AS}=B{\hat{\alpha }}_{AS}=\left[\hat{f}\left(s_{1}\right),\ldots ,\hat{f}\left(s_{n}\right)\right]{}^{\prime }.$$

From Equations (29) and (30), we can see that the fitted values of the dependent time series data can be obtained as:

(31) $${\hat{\mu }}_{AS}=\left(X{\hat{\beta }}_{AS}+{\hat{f}}_{AS}\right)=H_{AS}Y_{\hat{G}}=E\left[Y|X,\;s\right],$$

where $H_{AS}$ denotes the hat matrix, given by:

(32) $$H_{AS}=\left[X{\left[\left(X^{\prime }V-A_{AS}\right)X\right]}^{-1}\left(X^{\prime }-A_{AS}\right)V\left(I-M_{AS}\right)+M_{AS}\right],$$

with $M_{AS}=B{\left[B^{\prime }VB+\lambda K\right]}^{-1}B^{\prime }V^{\prime }$.

To make the computation process efficient, all penalty terms $\left(D^{T}WD\right)$ are calculated by using the iteration process instead of finding matrix D and knot set individually. The iterative algorithm is given in Algorithm 1 below.
**Algorithm 1**. Iterative algorithm process for the modified A-spline (AS) estimator ${\hat{\alpha }}_{AS}$.**Input**: X**,**s**,**$Y_{\hat{G}}.$**Output**: ${\hat{\beta }}_{AS}^{\left(i\right)}=\left({\hat{\beta }}_{1}^{\left(i\right)},{\hat{\beta }}_{2}^{\left(i\right)},\ldots ,{\hat{\beta }}_{p}^{\left(i\right)}\right)$   ${\hat{\alpha }}_{AS}^{\left(i\right)}={\left({\hat{\alpha }}_{1}^{\left(i\right)},{\hat{\alpha }}_{2}^{\left(i\right)},\ldots ,{\hat{\alpha }}_{q+k+1}^{\left(i\right)}\right)}^{\prime }$1: **Begin**2: Give initial values, $\beta^{\left(0\right)}=1_{p}$,  $\alpha^{\left(0\right)}=0_{q+k+1}$ and $W^{\left(0\right)}=I$ to start iterative process3: **do** until converges weighted differences to $L_{0}$-norm4: ${\hat{\beta }}_{AS}^{\left(i\right)}=({\left(X^{\prime }V-A)X\right)}^{-1}\left(X^{\prime }-A\right)VY_{\hat{G}}\;$5: ${\hat{\alpha }}_{AS}^{\left(i\right)}={\left[B^{\prime }VB+\lambda K\right]}^{-1}B^{\prime }V^{\prime }\left(Y_{\hat{G}}-X{\hat{\beta }}_{AS}^{\left(i\right)}\right)$6: Determine $\gamma =10^{-5}$7: $w_{i}^{\left(i\right)}={\left[{\left(\Delta^{q}\alpha_{i}^{\left(i\right)}\right)}^{2}+\gamma^{2}\right]}^{-1}$8: ${\hat{\beta }}_{AS}=\beta_{AS}^{\left(i\right)},\;\;{\hat{\alpha }}_{AS}={\hat{\alpha }}_{AS}^{\left(i\right)}$, **W** = *diag*$\left(w_{i}^{\left(i\right)}\right)$9: **end**10: Calculate $r_{\left(i^{*}\right)}$ by the criterion of ${\left(\Delta^{q}\alpha_{AS}^{\left(i\right)}\right)}^{2}W^{\left(i\right)}>0.99$11: Return ${\hat{\beta }}_{AS}^{\left(i\right)}=\left({\hat{\beta }}_{1},{\hat{\beta }}_{2},\ldots ,{\hat{\beta }}_{p}\right),$ ${\hat{\alpha }}_{AS}^{\left(i\right)}={\left({\hat{\alpha }}_{1},{\hat{\alpha }}_{2},\ldots ,{\hat{\alpha }}_{q+k+1}\right)}^{\prime }$12: **End**

**Remark** **2.** *For the constant value of* $\gamma =10^{-5}$ *, the iteration process repeats between step 3 and step 9 until the pre-determined tolerance value* $\delta =10^{-4}$ *is obtained where* $\delta =\overset{n}{\underset{i=1}{\sum }}n^{-1}\left|Y_{i}-{\hat{Y}}_{i\hat{G}}\right|$ *. From our experience, the expected number of iterations is observed as* no.iteration=20 *to achieve the convergence.*

Notice that the complexity and efficiency of Algorithm 1 is analyzed from different aspects that are given by:

(i) Number of local searches: algorithm does not involve a local search procedure which is an advantage for the speed of Algorithm 1;

(ii) Number of nested loops: due to the fact that there is only an iteration loop (without nested loops), if an algorithm does not include nested loops, its “order of growth” will be O(n);

(iii) Asymptotic behaviors: as the former inference mentioned, Algorithm 1 has O(n) which means that the limiting case of its convergence speed is considerable when it is compared with its alternative BS method on this issue.

As mentioned at the beginning of this section, the choice of an optimum smoothing parameter λ is required for both semiparametric BS and AS estimators. In this context, the improved Akaike information criterion ($AIC_{c})$ proposed by [29] is used, which is computed with the following equation:

(33) $$AIC_{c}\left(\lambda \right)=\log\left({\hat{\sigma }}^{2}\right)+1+\frac{2\left\{tr\left(H\right)+1\right\}}{n-tr\left(H\right)-2},$$

where ${\hat{\sigma }}^{2}$ is the estimate of the model variance, which is estimated for both methods separately in the next section, and H denotes the hat matrix for any of two methods. It is replaced by $H_{AS}$ for the AS method and $H_{BS}$ for the BS method, respectively.

## 4. Statistical Properties of the Estimators

In this paper, we introduced the semiparametric AS and BS estimators for the estimation of the right-censored time series model. It should be noted that these two methods were used for the first time in the setting of a time series estimation procedure. Inferences were therefore carried out about their statistical properties. For example, among these, the error terms obtained from the estimates of both methods and the estimators of parametric and nonparametric components were inspected and their properties were extracted.

### 4.1. Properties of the Semiparametric BS Estimator

Firstly, the parametric component was inspected. As is known, in a parametric context, errors can be decomposed into the bias and the variance terms that provide the quality of the estimator. Accordingly, the estimator ${\hat{\beta }}_{BS}$ of the parametric coefficients vector is expanded as follows:

(34) $${\hat{\beta }}_{BS}={\left[(X^{\prime }V-A_{BS})X\right]}^{-1}(X^{\prime }V-A_{BS})Y_{\hat{G}}=\beta +{\left[(X^{\prime }V-A_{BS})X\right]}^{-1}(X^{\prime }V-A_{BS})f,$$

where $V,\;A_{BS}$ and $M_{BS}$ matrices are as defined in Section 3.1 and $f={\left[f\left(s_{1}\right),f\left(s_{2}\right),\ldots ,f\left(s_{n}\right)\right]}^{\prime }$. From here, bias $B\left({\hat{\beta }}_{BS}\right)$ and variance-covariance $V\left({\hat{\beta }}_{BS}\right)$ of estimator ${\hat{\beta }}_{BS}$ can be computed as follows:

(35) $$B\left({\hat{\beta }}_{BS}\right)=E\left({\hat{\beta }}_{BS}\right)-\beta ={\left[(X^{\prime }V-A_{BS})X\right]}^{-1}(X^{\prime }V-A_{BS})f,$$

(36) $$V\left({\hat{\beta }}_{BS}\right)=\sigma^{2}{\left[(X^{\prime }V-A_{BS})X\right]}^{-1}(X^{\prime }V-A_{BS})X{\left[(X^{\prime }V-A_{BS})X\right]}^{-1},$$

where $\sigma^{2}$ is the variance of the fitted semiparametric model. Since the variance is not generally known, instead of $\sigma^{2}$, the estimation ($\mathrm{denoted}\;\mathrm{by}\;{\hat{\sigma }}_{BS}^{2}$) based on the BS is used. It can be computed from the residuals sum of squares (RSS) using error terms:

(37) $${\hat{\sigma }}_{BS}^{2}=\frac{RSS}{tr{\left(I-H_{BS}\right)}^{2}}=\frac{{\left\|\left(I-H_{BS}\right){\hat{Y}}_{{\hat{G}}_{BS}}\right\|}^{2}}{tr\left[{\left(I-H_{BS}\right)}^{\prime }\left(I-H_{BS}\right)\right]},$$

where $tr{\left(I-H_{BS}\right)}^{2}=n-2tr\left(H_{BS}\right)+tr\left(H_{BS}^{\prime }H_{BS}\right)$ denotes the degrees of freedom. In addition, $tr\left(H_{BS}^{\prime }H_{BS}\right)$ needs O(n) algebraic operations. In the context of the BS, if the data have a normal distribution, ${\hat{\sigma }}_{BS}^{2}$ is asymptotically unbiased.

Secondly, the properties of estimated nonparametric component ${\hat{\alpha }}_{BS}={\left({\hat{\alpha }}_{1},{\hat{\alpha }}_{2},\ldots ,{\hat{\alpha }}_{q+k+1}\right)}^{\prime }$ are given here. The bias of $\hat{\alpha }$ is one of the quality measurements for the estimated model. The bias is denoted as conditional expectation $E\left[\hat{\alpha }|s_{t}\right]$, given by:

(38) $$E\left[{\hat{\alpha }}_{BS}|s_{t}\right]={\left(B^{\prime }VB+\lambda D^{\prime }D\right)}^{-1}B^{\prime }VB\alpha .$$

From that, the bias is given by:

(39) $$Bias\left({\hat{\alpha }}_{BS}\right)\;=\;E\left[{\hat{\alpha }}_{BS}|s_{t}\right]-\alpha \;=\;{\left[\left(B^{\prime }VB+\lambda D^{\prime }D\right)\right]}^{-1}B^{\prime }V^{\prime }f-{\left[\left(B^{\prime }VB+\;\lambda D^{\prime }D\right)\right]}^{-1}B^{\prime }V^{\prime }X{\left[(X^{'}V-A_{BS})X\right]}^{-1}(X^{'}V-A_{BS})-{\left[\left(B^{\prime }VB+\lambda D^{\prime }D\right)\right]}^{-1}B^{\prime }V^{\prime }\;=\;\;{\left[\left(B^{\prime }VB+\lambda D^{\prime }D\right)\right]}^{-1}B^{\prime }V^{\prime }X{\left[(X^{'}V-A_{BS})X\right]}^{-1}(X^{'}V-A_{BS}).$$

Accordingly, the covariance of ${\hat{\alpha }}_{BS}$ can be computed as:

(40) $$Cov\left({\hat{\alpha }}_{BS}\right)={\hat{\sigma }}_{BS}^{2}\frac{1}{n}{\left(B^{\prime }VB+\lambda D^{\prime }D\right)}^{-1}\left(B^{\prime }VB\right){\left(B^{\prime }VB+\lambda D^{\prime }D\right)}^{-1},$$

where ${\hat{\sigma }}_{BS}^{2}$ is defined by Equation (36). In addition, to reveal the performance of ${\hat{f}}_{BS}=B{\hat{\alpha }}_{BS}$, the root square of mean squared error $RMSE\left(f,{\hat{f}}_{BS}\right)$ is used:

(41) $$RMSE\left(f,{\hat{f}}_{BS}\right)=n^{-1}\sum_{t=1}^{n}{\left[f\left(s_{t}\right)-{\hat{f}}_{BS}\left(s_{t}\right)\right]}^{2}=n^{-1}{\left(f-{\hat{f}}_{BS}\right)}^{\prime }\left(f-{\hat{f}}_{BS}\right).$$

### 4.2. Properties of the Semiparametric AS Estimator

Similar to in Section 4.1, the same properties for parametric and nonparametric components are given for the AS estimator here. The necessary expansion is written as follows to derivate the bias and variance of ${\hat{\beta }}_{AS}$:

(42) $${\hat{\beta }}_{AS}={\left[(X^{\prime }V-A_{AS})X\right]}^{-1}(X^{\prime }V-A_{AS})Y_{\hat{G}}\;=\beta +{\left[(X^{\prime }V-A_{AS})X\right]}^{-1}(X^{\prime }V-A_{AS})f,$$

where $A_{AS}$ and $M_{AS}$ are given in Section 3.2. Now, the bias and the covariance matrix of the estimator ${\hat{\beta }}_{AS}$ can be provided by:

(43) $$B\left({\hat{\beta }}_{AS}\right)=E\left({\hat{\beta }}_{A}\right)-\beta ={\left[(X^{\prime }V-A_{AS})X\right]}^{-1}(X^{\prime }V-A_{AS})f,$$

(44) $$V\left({\hat{\beta }}_{AS}\right)=\sigma^{2}{\left[(X^{\prime }V-A_{AS})X\right]}^{-1}(X^{\prime }V-A_{AS})X{\left[(X^{\prime }V-A_{AS})X\right]}^{-1},$$

where $\sigma^{2}$ is the variance of the fitted semiparametric model. Similar to Equation (40), instead of the model variance,$\;{\hat{\sigma }}_{AS}^{2}$ is obtained as follows:

(45) $${\hat{\sigma }}_{AS}^{2}=\frac{RSS}{tr{\left(I-H_{AS}\right)}^{2}}=\frac{{\left\|\left(I-H_{AS}\right){\hat{Y}}_{{\hat{G}}_{AS}}\right\|}^{2}}{tr\left[{\left(I-H_{AS}\right)}^{\prime }\left(I-H_{AS}\right)\right]}.$$

The properties of estimated nonparametric component ${\hat{\alpha }}_{AS}={\left({\hat{\alpha }}_{1},{\hat{\alpha }}_{2},\ldots ,{\hat{\alpha }}_{q+k+1}\right)}^{\prime }$ for the AS method are described below. The bias and the variance of the AS estimator ${\hat{\alpha }}_{AS}$ can be given, respectively, as:

(46) $$Bias\left({\hat{\alpha }}_{AS}\right)\;=\;E\left[{\hat{\alpha }}_{AS}|s_{t}\right]-\alpha \;=\;{\left[\left(B^{\prime }VB+\lambda D^{\prime }WD\right)\right]}^{-1}B^{\prime }V^{\prime }f-{\left[\left(B^{\prime }VB+\;\lambda D^{\prime }WD\right)\right]}^{-1}B^{\prime }V^{\prime }X{\left[(X^{'}V-A_{AS})X\right]}^{-1}(X^{'}V-A_{AS})-{\left[\left(B^{\prime }VB+\;\lambda D^{\prime }WD\right)\right]}^{-1}B^{\prime }V^{\prime }f\;=\;{\left[\left(B^{\prime }VB+\lambda D^{\prime }WD\right)\right]}^{-1}B^{\prime }V^{\prime }X{\left[(X^{'}V-A_{AS})X\right]}^{-1}(X^{'}V-A_{AS}),$$

and

(47) $$Cov\left({\hat{\alpha }}_{AS}\right)={\hat{\sigma }}_{AS}^{2}\frac{1}{n}{\left(B^{\prime }VB+\lambda D^{\prime }WD\right)}^{-1}\left(B^{\prime }VB\right){\left(B^{\prime }VB+\lambda D^{\prime }WD\right)}^{-1}.$$

Thus, the value of $RMSE\left(f,{\hat{f}}_{AS}\right)$ for ${\hat{f}}_{AS}=B{\hat{\alpha }}_{AS}$, similar to Equation (41), is calculated as follows:

(48) $$RMSE\left(f,{\hat{f}}_{AS}\right)=n^{-1}\sum_{t=1}^{n}{\left[f\left(s_{t}\right)-{\hat{f}}_{AS}\left(s_{t}\right)\right]}^{2}=n^{-1}{\left(f-{\hat{f}}_{AS}\right)}^{\prime }\left(f-{\hat{f}}_{AS}\right).$$

### 4.3. Quality Measures for the Fitted Model

After assessing the parametric and nonparametric components of the model in Section 4.1 and Section 4.2, several measurements are introduced in this section to evaluate the overall model performance. In the literature on time series modelling, mean absolute percentage error (MAPE), mean absolute error (MAE), and mean squared error (MSE) are the most commonly used performance criteria. To represent these criteria, MAPE is preferred in this study. In addition, median absolute error (MedAE) was used, which allowed us to account for missing or censored data. Generalized MSE (GMSE) and the ratio of GMSE (RGMSE) proposed by [30] and [2], respectively, were used to measure the quality of the fitted time series model. The aforementioned criteria can be defined as follows:

$$\begin{array}{cc} MAPE\left(Y_{t\hat{G}},{\hat{Y}}_{t\hat{G}}\right)=\frac{n^{-1}\sum_{t=1}^{n}\left|Y_{t}-{\hat{Y}}_{t\hat{G}}\right|}{Y_{t\hat{G}}}, & MedAE\left(Y_{\hat{G}},{\hat{Y}}_{\hat{G}}\right)=Median\left(\left|Y_{\hat{G}}-{\hat{Y}}_{\hat{G}}\right|\right), \end{array}$$

$$GMSE\left(Y_{\hat{G}},{\hat{Y}}_{\hat{G}}\right)={\left(Y_{\hat{G}}-{\hat{Y}}_{\hat{G}}\right)}^{\prime }E\left(Y_{\hat{G}}Y_{\hat{G}}^{\prime }\;\right)\left(Y_{\hat{G}}-{\hat{Y}}_{\hat{G}}\right),$$

where ${\hat{Y}}_{t\hat{G}}$ and ${\hat{Y}}_{\hat{G}}$ denote the fitted dependent variable values and vector for any estimation method. Here, ${\hat{Y}}_{t\hat{G}}$ and ${\hat{Y}}_{\hat{G}}$ are replaced by ${\hat{Y}}_{t{\hat{G}}_{BS}}$ and ${\hat{Y}}_{{\hat{G}}_{BS}}$ for the BS, and ${\hat{Y}}_{t{\hat{G}}_{A}}$ and ${\hat{Y}}_{{\hat{G}}_{A}}$ for the AS. In addition, to make a more considerable comparison between the AS and BS estimators, RGMSE is defined below.

**Definition** **2.** *The ratio of GMSE can be defined as follows:*

(49) $$RGMSE\left(Y_{{\hat{G}}_{BS}},{\hat{Y}}_{{\hat{G}}_{AS}}\right)=\frac{GMSE\left({\hat{Y}}_{{\hat{G}}_{AS}}\right)}{GMSE\left({\hat{Y}}_{{\hat{G}}_{BS}}\right)}.$$

Regarding the RGMSE criterion, if $\mathrm{RGMSE}\;\left(Y_{{\hat{G}}_{\mathrm{BS}}},{\hat{Y}}_{{\hat{G}}_{\mathrm{AS}}}\right)<1$, then it can be seen that the fitted model by the AS method shows better performance then the BS method.

## 5. Further Information for Adaptive-Ridge Penalty

The semiparametric AS estimator proposed for the right-censored time series model, with its adaptive nature, aims for qualified estimations despite the censorship. To approach the $L_{0}$-norm given in Equation (23), the most suitable knot locations can be chosen due to the weighted penalty term. Thus, the model avoids the disadvantages of synthetic data transformation, which gives higher magnitudes to uncensored observations.

This section is designed to inspect some of the large sample properties of the modified AS estimator under right-censored data. It should be noted that adaptive ridge penalty in the setting of regression has been studied by many authors; see for example [25,26,28]. However, the aforementioned studies consider adaptive ridge penalty individually, not as a part of a semiparametric time series model. This section provides basic information for the large sample properties of the proposed AS estimator in the context of a semiparametric time series model.

As previously stated, the AS approximation is a modified version of the P-splines (penalized BSs) estimator proposed by [31]. Note also that the AS method diverges from BSs with a significant difference of the $L_{0}$-norm in the penalty term. The AS estimator is obtained by an iterative process with determining weights, as expressed in Section 3.2. In addition, apart from the usage of the AS method in the literature, it is also used for modelling censored time series. For these reasons, we can make several important assumptions. The large sample properties are written based on the assumptions given below:

**Assumption** **1.** *The minimization problem for the semiparametric AS is given in Equation (26). To make this expression more general, it can be rewritten as follows:*

(50) $$PSS\left(\alpha ;\lambda \right)=\overset{n}{\underset{t=1}{\sum }}V{\left\{Y_{t\hat{G}}-\overset{p}{\underset{l=1}{\sum }}x_{tl}\beta_{l}-\overset{v}{\underset{j=1}{\sum }}\alpha_{j}B_{j,q}(s_{t})\right\}}^{2}+\lambda \overset{q+k+1}{\underset{j=q+1}{\sum }}{\left\|\Delta^{q}\alpha_{j}\right\|}_{\tau }{,}_{\;}\;$$

*where*${\left\|\Delta^{q}\alpha_{j}\right\|}_{\tau }$*represents the*τ*-norm of the penalty term. The first assumption is*τ→0*, which allows approximation to the*$L_{0}$*-norm with the acquisition of weights via the iterative process. Otherwise, the*$L_{0}$*-norm needs overly complex calculations, which leads to the loss of practicality when using the method. From our knowledge of the literature, when*τ→0,*such as in Equation (26), the minimization of Equation (50) works by penalizing the non-zero coefficients*$\alpha_{j}$*’s, as shown by [32]*.

**Assumption** **2.** *When*${\hat{\alpha }}_{AS}$*is examined asymptotically, the objective function of Equation (26) may not have a global minimum, since it is not clearly convex. However, if we assume that:*

(51) $$R_{n}=\frac{1}{r}\sum_{i}^{r}B_{i}B_{i}^{.{}^{\prime }}\to R,$$

*then it is possible to point out some important aspects of asymptotic consistency. Therefore, it should be presumed that*R*is a non-negative definite matrix and:*

(52) $$\frac{1}{q+k+1}\underset{1\leq i\leq r}{max}B_{i}^{\prime }B_{i}\to 0,$$

*where elements of*$diag\left(R_{i}\right)=1$.

**Assumption** **3.**
$B_{j}^{T}B_{j}$ *,* ${\left(B_{j}^{T}B_{j}\right)}^{-1}$ *, and* R *are assumed to be full rank matrices. Under the assumptions given above, to see asymptotic consistency of* ${\hat{\alpha }}_{AS}$ *and* ${\hat{\beta }}_{AS}$ *, an equation can be obtained from Equation (50) as follows:*

(53) $$M_{n}\left({\hat{\alpha }}_{ASn},{\hat{\beta }}_{ASn}\right)=\sum_{t=1}^{n}\begin{array}{c} V{\left\{Y_{t\hat{G}}-\overset{p}{\underset{l=1}{\sum }}x_{tl}{\hat{\beta }}_{ASnl}-\overset{r}{\underset{j=1}{\sum }}{\hat{\alpha }}_{ASnj}B_{j,q}(s_{t})\right\}}^{2}\\ +\lambda_{n}\overset{q+k+1}{\underset{i=q+1}{\sum }}{\left\|\Delta^{q}{\hat{\alpha }}_{ASn_{i}}\right\|}_{\tau }\;\; \end{array},$$

*where* $\left({\hat{\alpha }}_{ASn},{\hat{\beta }}_{ASn}\right)\;$ *denote the limiting case of the estimators for* $\lambda_{n}=O\left(n\right)$ *. Note that Equation (52) is ensured by following Theorem 1.*

**Theorem** **1.** *Based on Assumptions 1–3, and* $\lambda_{n}\to \lambda \geq 0$ *, then* $\left({\hat{\beta }}_{AS_{n}},\;{\hat{\alpha }}_{AS_{n}}\right)\overset{d}{\to }argmin\left(M_{n}\right)$ *where:*

(54) $$M_{n}\left({\hat{\beta }}_{AS_{n}},\;{\hat{\alpha }}_{AS_{n}}\right)={\left[{\left({\hat{\beta }}_{AS_{n}}\;\;{\hat{\alpha }}_{AS_{n}}\right)}^{\prime }-{\left(\beta \;\;\alpha \right)}^{\prime }\right]}^{\prime }R\left[{\left({\hat{\beta }}_{AS_{n}}\;\;{\hat{\alpha }}_{AS_{n}}\right)}^{\prime }-{\left(\beta \;\;\alpha \right)}^{\prime }\right]+\;\lambda_{n}\sum_{i=q+1}^{m=q+k+1}{\left\|\Delta^{q}\alpha_{i}\right\|}_{\tau }.$$

*Therefore, for optimal*$\lambda_{n}=O\left(1\right)$*, pair*$\left({\hat{\beta }}_{AS_{n}},\;{\hat{\alpha }}_{AS_{n}}\right)$*can be counted as a consistent AS estimator of*(β, α)*. In this context, when*n→∞*then*$\left|{\hat{\beta }}_{AS_{n}},\;{\hat{\alpha }}_{AS_{n}}\right|\to \left(\beta ,\;\alpha \right)$.

For the proof of Theorem 1, see Appendix C.

To clearly indicate the place of Assumptions 1–3 in the estimation process, the following explanations are given for each assumption.

- Assumption 1 is independent from the data. We assume that to provide a practical solution when minimizing Equation (50). Therefore, in both empirical and real data studies, this assumption does not impose anything to the dataset, but it is necessary to reduce the computational complexity.
- In real data studies, to ensure Assumption 2, **“**B**”** matrix obtained by using the nonparametric covariate needs to have independent columns. Because $\left(B^{\prime }B\right)$ should be identifiable and avoid the ill-posed problem, $\left(B^{\prime }B\right)$ must be a full-ranked matrix.
- Assumption 3 confirms Assumption 2. Thus, it can be seen that asymptotic consistency can be confirmed by Assumption 3. From that it can be said that Assumption 3 is indirectly depended on the dataset.

### 5.1. Asymptotic Distribution and Consistency of the Proposed Estimator

In this section, the estimate of parametric component ${\hat{\beta }}_{AS}$ is inspected in terms of asymptotic consistency and asymptotic distribution.

Assume the following regularity conditions:

(i) $F_{n}=n^{-1}\left(X_{i}^{T}V-A\right)X_{i}\to F$ for non-negative matrix F;
(ii) $n^{-1}\underset{1\leq t\leq n}{\max}\left(X_{i}^{T}V-A\right)X_{i}\;\to 0;$
(iii) Autoregressive errors $\varepsilon_{t}$’s given in Equation (2) are stationary with independent and identically distributed random error terms $u_{t}$’s that have zero mean and finite variance $0<\sigma^{2}<\infty$;
(iv) $F_{n}^{-1}=n^{-1}{\left[\left(X_{i}^{T}V-A\right)X_{i}\right]}^{-1}$ exists.

Here, condition (ii) indicates that the diagonal elements of F and $F_{n}$ are identical and one, because the covariates are scaled. To obtain the asymptotic distribution of ${\hat{\beta }}_{AS}$, “nearly-singular” designs are performed due to τ→0 for $F_{n}$. Thus, it can be ensured that $F_{n}\to F$ asymptotically. On the other hand, $F_{n}$ and F are assumed as non-singular in Section 5.1.

To show the consistency and asymptotic normality of the semiparametric AS estimator when conditions (i), (ii), and (iii) are ensured with non-singular F, first the case of τ≥1 is considered, followed by the case of τ<1.

Let ${\hat{\beta }}_{AS_{n}}$ be an asymptotic estimator. The consistency of ${\hat{\beta }}_{AS_{n}}$ can be reached by using following minimization function:

(55) $$\psi_{n}\left({\hat{\beta }}_{AS_{n}},\hat{f}\left(s_{t}\right)\right)=n^{-1}\sum_{t=1}^{n}{\left[Y_{t}-X_{t}{\hat{\beta }}_{AS_{n}}-\hat{f}\left(s_{t}\right)\right]}^{2}+\lambda_{n}n^{-1}\sum_{j=1}^{p}{\left|{\hat{\beta }}_{\left(j\right)AS_{n}}\right|}^{\tau }.$$

The following theorem shows the consistency of ${\hat{\beta }}_{AS_{n}}$ for validated additional assumption $\lambda_{n}=O\left(n\right)$.

**Theorem** **2.** *Assume that* F *is non-singular,* $\hat{f}\left(s_{t}\right)$ *behaves stable, and* $\lambda_{n}n^{-1}\to \lambda_{0}\geq 0$ *. It can then be said that as* n→∞ *:*

(56) $${\hat{\beta }}_{AS_{n}}\overset{d}{\to }\beta ,$$

*where* ${\hat{\beta }}_{AS_{n}}$ *is a consistent estimator of* β *. The proofs of this theorem are given in Appendix D. For* $\lambda_{n}=O\left(n\right)$ *,* argmin(ψ)=β *and therefore* ${\hat{\beta }}_{AS_{n}}$ *is a consistent estimator.*

It should be emphasized that the consistency of ${\hat{\beta }}_{{\mathrm{AS}}_{n}}$ is sufficient to show that $\lambda_{n}=O\left(n\right)$. However, this depends on the magnitude of growth of $\lambda_{n}$. When $\lambda_{n}$ grows more slowly, then a limiting distribution $\sqrt{n}\left({\hat{\beta }}_{{\mathrm{AS}}_{n}}-\beta \right)$ exists. It is clear from Theorem 2 that the mean of the limiting distribution of $\sqrt{n}\left({\hat{\beta }}_{{\mathrm{AS}}_{n}}-\beta \right)$ converges to zero for the consistency of ${\hat{\beta }}_{{\mathrm{AS}}_{n}}$. In addition, its asymptotic variance can be obtained based on conditions (i) and (iv) as $\sigma^{2}F^{-1}$. Accordingly, the asymptotic distribution of the semiparametric AS estimator is written as:

(57) $$\theta =\sqrt{n}\left({\hat{\beta }}_{{\mathrm{AS}}_{n}}-\beta \right)\overset{d}{\to }N\left[0,{\;\sigma }^{2}F^{-1}\right].$$

However, the limiting distribution depends on whether τ<1 or τ≥1. In the context of this paper, Theorem 3 is given for the limiting distribution of ${\hat{\beta }}_{{\mathrm{AS}}_{n}}$ when τ<1.

**Theorem** **3.** *Assume that* τ<1 *if* $\lambda_{n}/n^{\frac{\tau }{2}}\to \lambda_{0}\geq 0$ *. Then:*

(58) $$\theta =\sqrt{n}\left({\hat{\beta }}_{AS_{n}}-\beta \right)\overset{d}{\to }argmin\left(\xi \right),$$

*where* $\xi \left(\theta \right)=-2\theta^{T}F+\theta^{T}F\theta +\lambda_{0}\overset{p}{\underset{j=1}{\sum }}{\left\|\theta_{j}\right\|}^{\tau }I\left(\beta_{j}=0\right)$ *. The proofs of Theorem 3 are given in Appendix E.*

## 6. Simulation Study

In this section, a simulation study was conducted to inspect the finite-sample behaviors and performances of the two semiparametric estimators $\left({\hat{\alpha }}_{BS},{\hat{\beta }}_{BS}\right)$ and $\left({\hat{\alpha }}_{AS},{\hat{\beta }}_{AS}\right)$ under right-censored time series. These estimators were then compared through the quality measurements given in Section 4. The simulation scenarios are designed as follows:

(a) We use the model $Z_{t}=X_{t}\beta +f\left(s_{t}\right)+\varepsilon_{t},\;t=1,2,\ldots ,n$ to generate datasets in the simulation experiments.
(b) The unknown smooth regression function $f\left(s_{t}\right)$ is constructed by combining the functions $\left\{S_{j},j=1,\ldots ,5\right\}$ that denote seasonal effects on the data, that is, $f\left(s_{t}\right)=U_{j=1}^{5}S_{j}\left(s_{i}\right)$, where $S_{j}\left(s_{i}\right)\;=\;s_{i}\sin^{2}\left(s_{i}\right)$ with $s_{i}=\frac{\left(i-0.5\right)}{\frac{n}{5}},i\;=1,\ldots ,\left(n/5\right).$
(c) The design matrix is generated from a normal distribution: $X_{t}\sim N\left(\mu_{x}=5,\;\sigma_{x}^{2}=1\right)$, where $X_{t}$ is the (n×p) dimensional matrix for p=3. Note also that the distribution may not be normal, and that one can thus consider a uniform or other distributions. The vectors of the regression coefficients are β=(3, 0.5,−1).
(d) The autoregressive error terms are generated from a one-lagged process $\varepsilon_{t}=\rho \varepsilon_{t-1}+u_{t}$ with ρ=0.5 and $u_{t}\sim N\left(0,1\right)$.
(e) Thus, as stated in (a), completely observed dependent time series $Z_{t}$’s are generated from the sum of the parametric, nonparametric, and error terms using (b), (c), and (d).
(f) To produce the right censored variable $Y_{t}$, as specified in Equation (3), we generate the censoring variable $C_{t}$ from the binomial distribution with proportions or censoring levels (CLs) at 5%, 20%, and 40%. The Algorithm 2, given below, demonstrates how the censoring variable is created.

**Algorithm 2**. Generation of censoring variable $C_{t}$.**Input:** Completely observed $Z_{t}$
**Output:** Right-censored dependent variable $Y_{t}$1: For given censoring level (CL), produce $\delta_{t}=I\left(Z_{t}\leq C_{t}\right)$ from the binomial distribution2: **for** (t in 1 to n)3:     **If** $\left(\delta_{t}=0\right)$4:        **while** $\left(Z_{t}\leq C_{t}\right)$5:        generate $C_{t}\sim N\left(\mu_{Z},\sigma_{Z}^{2}\right)$6:     **Else**7:           $C_{t}=Z_{t}$8: **end** (for loop in step 2)9: **for** (t in 1 to n)10:    **If**
$\left(Z_{t}\leq C_{t}\right)$11:         $Y_{t}=Z_{t}$12:    **Else**13:         $Y_{t}=C_{t}$
14: **end** (for loop in Step 9)

(a) To deal with censored observations in $Y_{t}$ obtained with Algorithm 2, we use synthetic data values $Y_{t\hat{G}}$ obtained through the Kaplan and Meier estimator [18], as described in Equation (6).
(b) AR(1) model is used as a naïve model to estimate the right-censored time-series as in [1,2]. Thus, the finite sample performance of the introduced methods can be made.

For each CL in the simulation experiments, we generated 1000 random samples for size n=50, 100, and 200.

**Figure 1:**
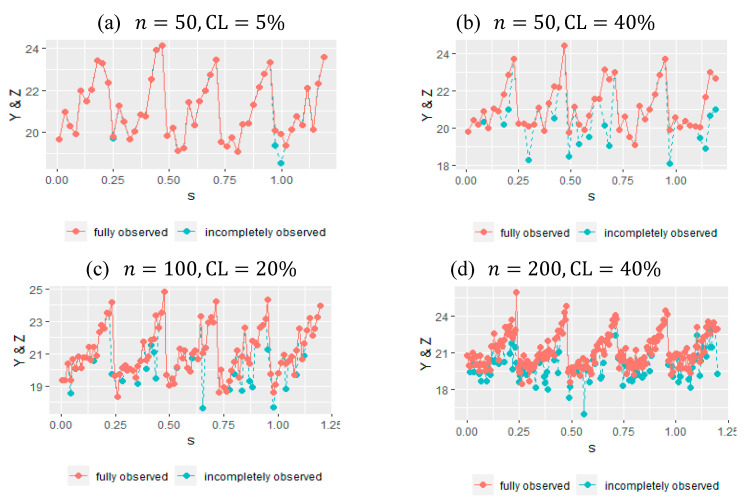
Some of the datasets generated using Algorithm 2 including both fully observed and censored data points for different censoring levels and sample sizes.

The results of the simulation study were divided into three parts for parametric components, nonparametric components, and overall model performance. Accordingly, the outcomes of the estimated models, comparative results, and corresponding comments are given together in the following tables and figures. To understand the simulated datasets and the scenarios, examples of some of the simulation configurations are given in Figure 1. Panel (a) shows the dataset for small sample size and low censorship. Panel (b) is drawn to show the case when the censoring level is really high. Panels (c)–(d) indicates the cases for medium and large sample sized data with censoring levels 20% and 40% respectively.

### 6.1. Assessing the Parametric Component

**Figure 2:**
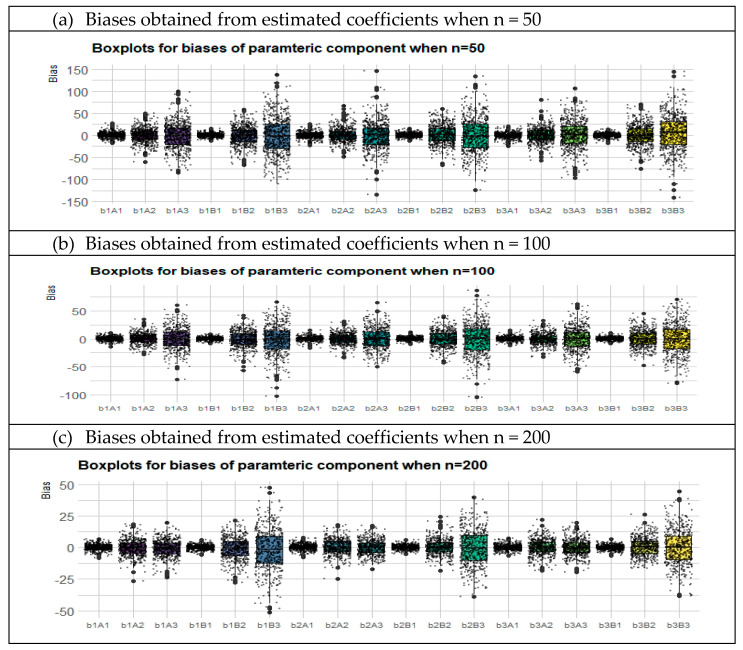
Boxplots of bias values for both the AS and BS methods for all configurations. In the x-axis, b1, b2, and b3 denote $\beta_{1},\;\beta_{2}$, and $\beta_{3}$; A1, A2, and A3 denote biases obtained from the AS method for CLs of 5%, 20%, and 40%. Similarly, B1, B2, and B3 denote biases for the BS method, when CLs are 5%, 20%, and 40%.

In this section, the performances of the two methods were compared in terms of the parametric components of the right-censored semiparametric linear models generated by the simulation. It should be also noted that in this simulation study, 54 different configurations were analyzed to provide a broad perspective of the adequacy of each method. The results from the parametric components in the simulation study are displayed in Table 1 and Figure 2. Note that bold colored scores indicate the best (minimum) scores.

From the careful inspection of Table 1, it can be demonstrated that the behaviors of the BS and AS change noticeably in different scenarios. Let us look at low and medium CLs for n=50; under these conditions, the BS has remarkable superiority over the AS. This can be interpreted as the BS fitting the data better when the data’s structure is distorted less by censorship. However, for CL=40%, which means the data are heavily censored, the AS method gives better scores.

As the sample size increases, although the bias and variance values from the methods are obtained more closely, the AS provides more efficient performance in estimating the parametric component. Regarding the parametric component, it should be emphasized that the AS behaves as expected and gives the best scores for cases of heavy censorship.

In general, the best scores for each method can be evaluated in terms of bias and variance results. When we examined the bias results of the regression coefficients, the AS method gives the best score in only 12 out of 27 configurations while the BS method gives the best score in 15. However, regarding the variances, the AS gives the best score in 18 of 27 configurations, while the BS is superior in only 9 configurations. In Figure 2, Panels (a–c) shows the calculated biases for each simulation repetition for all cases when sample size is small, medium, and large.

### 6.2. Evaluating the Nonparametric Component

As in the case of parametric components, we constructed 1000 estimates of the regression function f(.), which is the nonparametric component of model (1). For each method, 1000 replications were carried out, and the estimated bias, variance and *RMSE* values were computed for each estimator. This section is designed to show the simulated results related to the nonparametric component.

The results in Table 2 showed that the AS method proves its efficiency for the estimation of the nonparametric component when time series data are moderately to heavily censored. On the other hand, for CL=5%, the BS method gives better results for all sample sizes according to our evaluation metrics. One of the main reasons for this is that the BS adapted to the knots more than the AS. Consequently, when the data points are manipulated by censorship, these knots force the BS to make inefficient estimates. At this point, the knot determination of the AS based on the weights given in Equation (24) diminishes the effect of the censorship. That is why the AS method performs better under moderately and heavily censored time series data.

**Figure 3:**
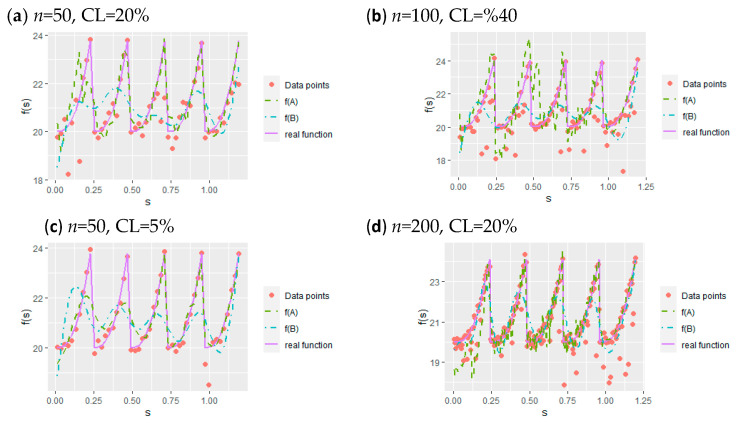
Data points, real regression functions, and curves fitted by two methods. In the legend of the plots, f(A) and f(B) represent function estimates obtained from the AS and BS methods, respectively.

Figure 3, consisting of four panels (a), (b), (c), and (d), is drawn to illustrate the performance of the AS and BS methods in nonparametric curve estimation and to present different simulation configurations. Panel (a) show the estimated curves for small sample size and medium censoring level. Similarly, Panel (b) shows the case when medium sample size and high censoring level. Panel (c) indicates the estimated curves for small sample size and low censoring level. Finally, Panel (d) shows the estimated curves when sample size is large and censoring level is medium. When panels (**a**) and (**c**) are analyzed comparatively, the effect of the censorship level can be seen. At the first glance, the distortion of both curves is noticeable. However, the BS method is insufficient to represent censored time series compared to the AS method. In addition, panel (b) shows that when data are heavily censored, the BS curve is drawn towards the x = 0 line, due to the presence of zero values in the synthetic response variable. Finally, panel (**d**) indicates that although the time series contains censored data points, the qualities of the estimates for both the AS and BS methods become better as the sample size increases.

### 6.3. Assessing the Performances of Methods

**Figure 4:**
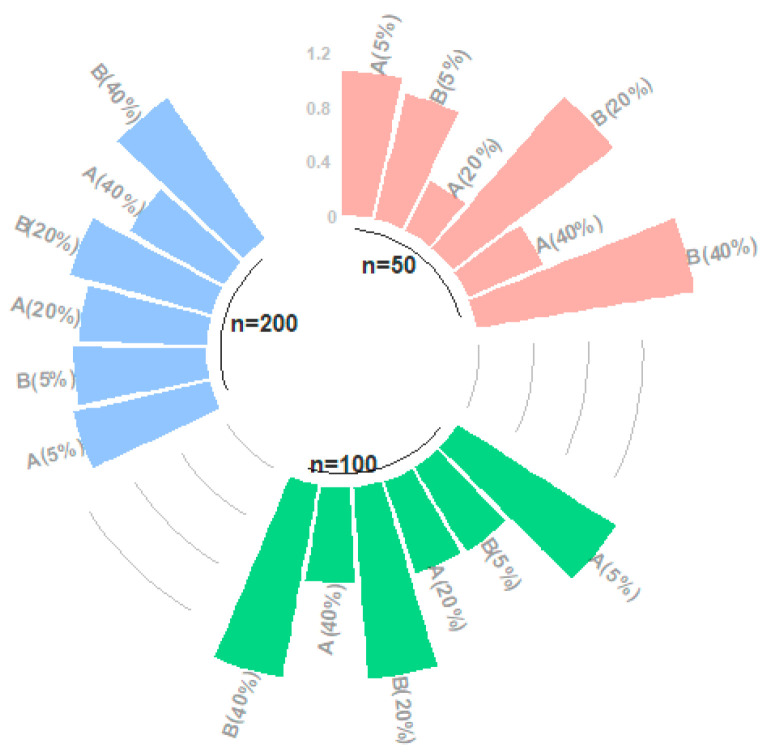
360° bar chart for the RGMSEs of all simulation combinations.

This section involves the results for overall model estimations obtained from the AS and BS methods. Although results are given for parametric and nonparametric components in the previous sections, a separate review for the whole model estimation is required for a healthy comparison. Accordingly, the performance scores for MAPE, MedAE, and GMSE are given in Table 3, and Figure 4 is drawn to illustrate the RGMSE values.

When Table 3 is examined, it can be seen that the results obtained for the model estimates are slightly different from the previous results, as expected. The total error obtained from the estimation of parametric and nonparametric components is one of the reasons for this discrepancy. In addition, considering the situations where the two methods produce extremely similar scores, this difference can be understood better. Note that AR(1) model shows poor performance, which depends on its parametric and linear structure. However, for the large sample size (n = 200), the scores of models obtained are close to each other. However, it is clearly seen that the AS and BS methods are much better on the estimation of right-censored time series.

As can be seen from the bolded scores, the AS method generally performs better. From Table 3, it can be seen that the MAPE values obtained by BS are better for n=50. However, as mentioned earlier, in this study, the MedAE criterion, which is not frequently used for time series data, is used to measure the durability of the predictions. When the scores of this criterion are examined, it is understood that, as stated from the beginning of the study, the BS method has more successful estimates under low censorship levels, but the AS method is superior for medium and high censorship levels.

Figure 4 includes the RGMSE scores for both the AS and BS methods that are formed by the ratio of the GMSE values of each method. In Figure 4, the difference between the qualities of the estimates is clearly very small for CL=5%. However, the difference becomes more significant for CL=20% and CL=40%. Note that for CL=5%, the BS method gives smaller ratio values, which confirms the results given in Table 3. As stated before, the AS method is demonstrably superior at higher censorship levels, which can be seen in Figure 4 for all sample sizes.

## 7. Real-World Data

**Figure 5:**
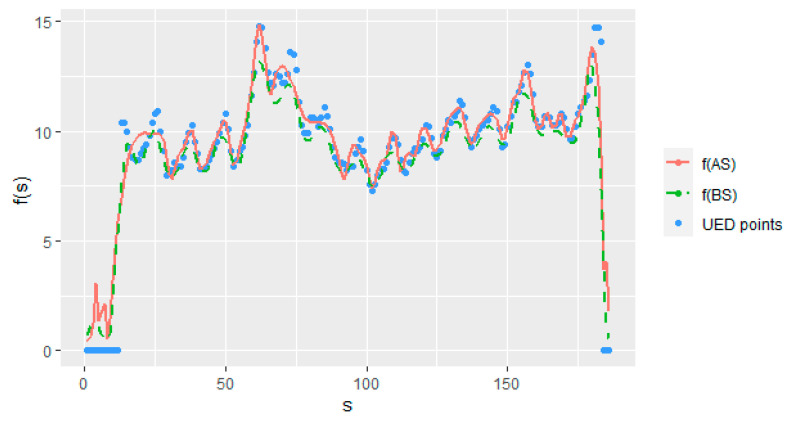
Estimated curves for the seasonality $f\left(s_{t}\right)$ obtained from the AS and BS methods.

This section is designed to show how the newly introduced semiparametric estimator AS and benchmark BS method behave with a real right-censored time series dataset. For this purpose, we consider unemployment duration data involving the monthly unemployment period rates years between 2004 and 2019 for Turkey; this dataset is available at https://ec.europa.eu/eurostat/databrowser/view/UNE_RT_M__custom_1635127/default/table?lang=en. In the dataset, the last three months of 2004 and the last three months of 2019 cannot be observed correctly. Therefore, these data points can be censored from the right by the detection limit zero, because none of the data points are negative values. Accordingly, the introduced semiparametric methods, AS and BS, can be used for this time series analysis. In addition, as in the simulation study, the results of the AR model are given in the following tables. However, different from the simulation study, AR(2) model was used for the real data study, because the optimal lag values is determined as lag=2 from Table 4. Before the modelling procedure, the stationarity of the time series data was tested with the augmented Dickey–Fuller (ADF) test, the suitable lag is determined under null hypothesis $H_{0}:y_{t}\;is\;non-stationary$. The test results are given in Table 4 below:

Table 4 shows that the second lag for this time series is suitable for the modelling. From this information, the semiparametric time series model can be given by:

(59) $$UED_{t}=\beta_{1}UED_{\left(t-1\right)}+\beta_{2}UED_{\left(t-2\right)}+f\left(s_{t}\right)+\varepsilon_{t},\;\;t=1,\ldots ,186,$$

where $UED_{t}$s represent the dependent time series of the unemployment duration ratio, $UED_{\left(t-1\right)}$ and $UED_{\left(t-2\right)}$ denote the first and second lags of the dependent series $UED_{t}$ that are used as covariates, respectively, $s_{t}={\left(1,\ldots ,n\right)}^{T}$ denotes the seasonality, and finally, $\varepsilon_{t}$’s are the stationary autoregressive error terms as given in Equation (2). The estimation of model (6.1) is realized by both the AS and BS methods, and then, results are presented in Table 5 and Table 6 and Figure 5.

Table 5 involves the bias and variance values for estimated regression coefficients $\hat{\beta }=\left({\hat{\beta }}_{1},{\hat{\beta }}_{2}\right)$ and $\hat{\alpha }={\left({\hat{\alpha }}_{1},{\hat{\alpha }}_{2},\ldots ,{\hat{\alpha }}_{q+k+1}\right)}^{T}$. Accordingly, the AS method gives smaller bias and variance values than the BS method regarding $\hat{\beta }$. Moreover, the AS method has better bias values for $\hat{\alpha }$**,** but the BS method gives smaller variance values for $\hat{\alpha }$ than the AS method. In overview, the AS and BS methods give similar values, because the data properties are n=186 and CL=8.1%. Thus, it can be seen that the results of the unemployment duration data ensure the simulation outputs.

In addition, it should be noted that the outcomes obtained from estimated model (7.1) are given in Table 6 with RMSE scores for the estimated nonparametric function $f\left(s_{t}\right)$. Upon close inspection, it is obviously seen from the results that the AS method produces the best scores. It should be emphasized that the largest difference between the methods regarding performance criteria is in MedAE, which indicates the strength of the AS method under censorship. Table 6 indicates the results of AR(2) model that are worse than the results of the other two as in the simulation study. Note that because of the sample size of the real data of n=186 which is close to the simulation configurations when n=200, scores are relatively close to each other. Figure 5 is given to compare the AS and BS methods in representing data under censorship.

As can be seen in Figure 5, the estimated curves are quite similar due to the data properties of a large sample size and a low CL. The effect of synthetic data manipulation is obvious in the figure with zero values. Like the simulation study, the BS method is affected by these zero values more than the AS method. The reason for this is that the knots of the AS method are determined by iteratively calculated weights. Therefore, the optimal knot sequence diminishes the effect of censorship.

## 8. Concluding Remarks

This paper demonstrated the estimation of right-censored time series data using a newly introduced semiparametric AS estimator and making a comparison with the BS method as a benchmark. The results obtained from both a simulation study and a real data example proved that the introduced method (AS) achieves the superior modelling of right-censored time series data in a semiparametric context. Comparative outcomes also support that the AS method provides better performance scores over the BS method in most simulation configurations and the real data example. The most important factor in the success of the AS method is the adaptive nature of the method based on iteratively calculated weights. In the AS method, weights are responsible for determining and controlling the penalty term and for dependently obtaining the optimal knot points. Accordingly, our findings showed that the proposed method provides an advantage in modelling right-censored time series over the benchmark.

The simulation study examined the performance of the methods in three parts: the outcomes for the estimated parametric component (Table 1 and Figure 2), the nonparametric component (Table 2 and Figure 3), and the whole semiparametric model (Table 3 and Figure 4). The unemployment data estimation was evaluated for bias and variance (Table 5) using the criteria of MAPE, MedAE, GMSE, and RGMSE (Table 6). Given the outcomes of the simulation study and the real data example, our general and detailed conclusions are as follows:

- As expected, the estimation qualities for both the AS and BS methods change for different CLs and sample sizes. The performances of the methods are affected negatively by increasing CLs, and they give better results for larger sample sizes. This claim is seen clearly from Table 1, Table 2 and Table 3.
- When unemployment duration data were analyzed, it can be seen that the results agreed with the corresponding configuration (n=200;  CL=20%) of the simulation study.
- One of the striking results of this paper is that, as Table 1, Table 2 and Table 3 demonstrate, while the AS method gives worse results at low censorship levels than the BS method, it provides significantly better results at medium and high censorship levels. This conclusion proves the claim of the paper, which is that using the AS method reduces the effect of the data manipulation of synthetic data transformation.
- When all the results obtained from simulation and real data studies were inspected, the AS method gives better results than the BS method, except in the configurations for low CLs, which supports the targeted conclusion.
- Unemployment duration data were modelled by the BS and AS methods using two lagged parametric components and the seasonal effect as a nonparametric component. Table 5 and Table 6 show each method’s scores using four evaluation metrics, which indicate the superiority of the AS method. Figure 5 shows the estimated curves for both methods, which are similar. However, the estimated curves show that the AS method is less affected by zero values of synthetic data and thus gives more satisfying estimates for the right-censored time series model than the BS method.

Finally, as can be understood from the whole paper, the AS method is superior for estimating right-censored time series over the BS method in both theory and practice.

## Figures and Tables

**Table 1:** Estimated regression coefficients from the AS and the B-spline (BS) with values of variance and bias.

		*β*_1_ = 3				$\beta_{2}\;=\;0.5$				*β*_3_ = −1			
		$\mathrm{Bias}({\hat{\beta }}_{1})$		$\mathrm{Var}({\hat{\beta }}_{1})$		$\mathrm{Bias}({\hat{\beta }}_{2})$		$\mathrm{Var}({\hat{\beta }}_{2})$		$\mathrm{Bias}({\hat{\beta }}_{3})$		$\mathrm{Var}({\hat{\beta }}_{3})$	
*n*	*C.L.*	AS	BS	AS	BS	AS	BS	AS	BS	AS	BS	AS	BS
50	5	0.887	**0.870**	0.936	**0.842**	0.809	**0.786**	0.922	**0.845**	0.867	**0.837**	0.884	**0.804**
	20	**0.852**	0.895	**1.180**	1.290	**0.888**	0.892	**1.210**	1.358	0.963	**0.949**	**1.191**	1.336
	40	**0.999**	1.172	**1.455**	1.641	**0.916**	1.108	**1.431**	1.657	**0.946**	1.145	**1.453**	1.674
100	5	0.510	**0.470**	0.440	**0.425**	0.539	**0.434**	0.433	**0.422**	0.515	**0.467**	0.439	**0.431**
	20	**0.514**	0.610	**0.583**	0.609	**0.538**	0.579	**0.583**	0.609	**0.527**	0.599	**0.590**	0.618
	40	0.535	**0.433**	**0.619**	0.689	**0.525**	0.622	**0.619**	0.689	**0.535**	0.610	**0.629**	0.692
200	5	0.285	**0.271**	0.260	**0.253**	0.290	**0.272**	0.255	0.255	0.294	**0.271**	**0.252**	0.254
	20	**0.310**	0.324	**0.333**	0.355	0.311	**0.300**	**0.325**	0.351	0.304	**0.296**	**0.328**	0.353
	40	**0.314**	0.333	**0.338**	0.352	**0.321**	0.337	**0.332**	0.356	**0.307**	0.336	**0.332**	0.363

The bolded values indicate the best scores.

**Table 2:** Outcomes from the fitted nonparametric components.

		$\mathrm{Bias}(\hat{\alpha })$		$\mathrm{Var}(\hat{\alpha })$		$\mathrm{RMSE}(f,\hat{f})$	
*n*	*CLs*	AS	BS	AS	BS	AS	BS
50	5	1.085	**0.629**	0.048	**0.022**	1.135	**0.883**
	20	**1.128**	1.498	**0.066**	0.075	**1.099**	2.061
	40	**1.287**	2.510	**0.079**	0.095	**2.511**	3.127
100	5	0.961	**0.851**	**0.022**	0.025	0.824	**0.664**
	20	**1.040**	1.217	**0.030**	0.041	**1.255**	1.779
	40	**1.070**	1.302	**0.037**	0.070	**1.815**	2.331
200	5	0.891	**0.813**	0.009	**0.008**	0.670	**0.435**
	20	**0.928**	0.959	**0.013**	0.021	**1.547**	1.871
	40	**0.995**	1.070	**0.017**	0.028	**2.397**	2.882

The bolded values indicate the best scores.

**Table 3:** The values of performances from the AS and BS methods.

		*MAPE*			*MedAE*			*GMSE*		
*n*	*CL*s	AS	BS	AR(1)	AS	BS	AR(1)	AS	BS	AR(1)
50	5	0.166	**0.157**	0.322	0.419	**0.383**	0.999	3.119	3.510	4.915
	20	0.358	**0.348**	0.388	**0.737**	0.896	1.052	4.468	4.920	5.142
	40	**0.584**	0.688	1.980	**1.030**	1.519	1.971	7.762	9.542	10.751
100	5	**0.154**	0.186	0.303	0.323	**0.320**	0.860	1.001	0.928	3.614
	20	**0.333**	0.336	0.365	**0.668**	0.750	0.914	1.870	1.988	4.147
	40	**0.514**	0.528	1.476	**1.025**	1.831	1.891	3.663	4.182	6.798
200	5	0.111	**0.096**	0.283	0.264	**0.251**	0.717	0.983	**0.761**	1.935
	20	**0.312**	0.332	0.364	**0.552**	0.606	0.847	**2.065**	2.497	3.411
	40	**0.499**	0.508	0.654	**1.008**	1.086	1.501	**2.759**	2.816	3.131

The bolded values indicated the best scores.

**Table 4:** Augmented Dickey–Fuller (ADF) test results for the stationarity of time series data and the determination of the appropriate lag.

No. Lag	ADF Test Results	*p*-Value
0	−2.61	0.318
1	−3.27	0.077
2	**−3.52**	**0.041**
3	−3.33	0.066
4	−3.30	0.072

Bold scores are significant score for the 95% confidence level.

**Table 5:** The performances of the BS and AS methods for the estimation of both parametric and nonparametric components.

Measurement	Bias		Variance	
	AS	BS	AS	BS
${\hat{\beta }}_{1}$	**1.941**	2.682	**1.272**	1.703
${\hat{\beta }}_{2}$	**0.915**	1.139	**1.562**	1.624
$\hat{\alpha }$	**3.628**	4.566	0.067	**0.058**

The bolded values indicate the best scores.

**Table 6:** Scores of performance measures for the AS and BS methods obtained from the whole model estimation.

Method	*MAPE*	*MedAE*	*GMSE*	*RGMSE*	$\mathrm{RMSE}(f,\hat{f})$
AS	**0.623**	**0.510**	**1.275**	**0.824**	**1.154**
BS	1.315	1.166	1.546	1.212	1.385
AR(2)	1.856	4.506	3.702	2.775	-

The bolded values indicate the best scores.

## Data Availability

We consider unemployment duration data involving the monthly unemployment period rates years between 2004 and 2019 for Turkey; this dataset is available at https://ec.europa.eu/eurostat/databrowser/view/UNE_RT_M__custom_1635127/default/table?lang=en.

## Appendix A

**Proof** **of** **Lemma** **1.** Lemma 1 can be ensured based on the common censorship assumption that $Z_{t}$ and $C_{t}$ are independent. From that, the proof can be written as follows:

(A1) $$\begin{array}{c} E\left[Y_{tG}|x,s\right]\;=\;E\left[\frac{\delta_{t}Z_{t}}{1-G\left(Z_{t}\right)}|\;x,s\right]=E\left[\frac{\delta_{t}Z_{t}}{\bar{G}\left(Z_{t}\right)}|x,s\right]\;\;\;\;\;\;\;\;\;\;\;\;\;\;\;\;\;\;\;\;=\;E\left[\frac{I\left(Z_{t}\leq C_{t}\right)\min\left(Z_{t},C_{t}\right)}{\bar{G}\left[\min\left(Z_{t},C_{t}\right)\right]}|x,s\right]=\\ E\left[I\left(Z_{t}\leq C_{t}\right)\frac{Z_{t}}{\bar{G}\left(Z_{t}\right)}|x,s\;\right]\;=\;E\left[E\left[\frac{Z_{t}}{\bar{G}\left(Z_{t}\right)}I\left(Z_{t}\leq C_{t}\right)|x,s\right]|x,s\right]=E\left[\frac{Z_{t}}{\bar{G}\left(Z_{t}\right)}\;\bar{G}\left(Z_{t}\right)|x,s\right]=\\ E\left[Z_{t}|x,s\right]=x_{t}\beta +f\left(s_{t}\right) \end{array}$$

Thus, Lemma 1 is proven. Here, $\bar{G}\left(.\right)=1-G\left(.\right).$ Generally, distribution G(.) is unknown. Therefore, its Kaplan–Meier estimator $\hat{G}\left(.\right)$ is used instead of G(.), which is given in Equation (5). □

## Appendix B

Derivations of Equations (29) and (30).

To show the derivations of Equations (29) and (30), two equations obtained from Equation (27) are written as:

(A2) $$\left(X^{\prime }VX\right)\beta +X^{\prime }VB\alpha =X^{\prime }VY_{\hat{G}}\;\;\;\;\;\;B^{\prime }VX\beta +\left(B^{\prime }VB+\lambda K\right)\alpha =B^{\prime }VY_{\hat{G}}$$

From Equation (B1), ${\hat{\alpha }}_{AS}$ can be acquired by the algebraic operations:

(A3) $$\left(B^{\prime }VB+\lambda K\right)\alpha =B^{\prime }VY_{\hat{G}}-B^{\prime }VX\beta \;\;\;\;\;\left(B^{\prime }VB+\lambda K\right)\alpha =B^{\prime }V\left(Y_{\hat{G}}-X\beta \right).$$

Thus, if β is replaced by ${\hat{\beta }}_{AS}$, then ${\hat{\alpha }}_{AS}$ can be written as:

(A4) $${\hat{\alpha }}_{AS}={\left[B^{\prime }VB+\lambda K\right]}^{-1}B^{\prime }V^{\prime }\left(Y_{\hat{G}}-X{\hat{\beta }}_{AS}\right).$$

Therefore, Equation (27) can be derived. Accordingly, the derivation of ${\hat{\beta }}_{AS}$ can be obtained by using (B1):

(A5) $$\left(X^{\prime }VX\right)\beta +X^{\prime }VB\left[{\left[B^{\prime }VB+\lambda K\right]}^{-1}B^{\prime }V^{\prime }\left(Y_{\hat{G}}-X\beta \right)\right]=X^{\prime }VY_{\hat{G}},\;\left(X^{\prime }VX\right)\beta +X^{\prime }VB{\left[B^{\prime }VB+\lambda K\right]}^{-1}B^{\prime }V^{\prime }Y_{\hat{G}}-X^{\prime }VB{\left[B^{\prime }VB+\lambda K\right]}^{-1}B^{\prime }V^{\prime }X\beta =X^{\prime }VY_{\hat{G},}\;\left[\left(X^{\prime }VX\right)-X^{\prime }VB{\left[B^{\prime }VB+\lambda K\right]}^{-1}B^{\prime }V^{\prime }X\right]\beta =X^{\prime }VY_{\hat{G}}-X^{\prime }VB{\left[B^{\prime }VB+\lambda K\right]}^{-1}B^{\prime }V^{\prime }Y_{\hat{G}}.$$

To simplify the calculations, let $A_{AS}=X^{\prime }VB{\left[B^{\prime }VB+\lambda K\right]}^{-1}B^{\prime }V^{\prime }$. Therefore,

(A6) $$\left[\left(X^{\prime }V-A_{AS}\right)X\right]\beta =\left(X^{\prime }-A_{AS}\right)VY_{\hat{G}},\;{\hat{\beta }}_{AS}=({\left(X^{\prime }V-A_{AS})X\right)}^{-1}\left(X^{\prime }-A_{AS}\right)VY_{\hat{G}}.$$

The derivations of Equations (29) and (30) are thus completed.

## Appendix C

**Proof** **of** **Theorem** **1.** To validate the Theorem 1, necessary equations are given by:

(A7) $$\underset{{\hat{\alpha }}_{ASn}\in Q}{\sup}\left|M_{n}\left({\hat{\alpha }}_{n}\right)-M\left({\hat{\alpha }}_{ASn}\right)-\sigma_{\varepsilon }^{2}\right|\overset{p}{\to }0,$$

where $\sigma_{\varepsilon }^{2}$ is the variance of the model defined in Equation (7), Q is a compact set in a metric space and by using Equations (54)–(57), it can be seen that:

(A8) $$\left|{\hat{\alpha }}_{ASn}\right|\to \alpha ,\;\mathrm{as}\;n\to \infty .$$

See [33] for more details. □

## Appendix D

**Proof** **of** **Theorem** **2.** For ensured regularity conditions (i)–(iv), $plim\left({\hat{\beta }}_{AS_{n}}\right)$ is written as follows:

(A9) $$plim\left({\hat{\beta }}_{AS_{n}}\right)\;=\beta +plim\left(n^{-1}{\left[(X^{\prime }V-A_{AS})X\right]}^{-1}(X^{\prime }V-A_{AS})f\right)+plim\left(n^{-1}{\left[(X^{\prime }V-\;A_{AS})X\right]}^{-1}(X^{\prime }V-A_{AS})\varepsilon \right)\;\;\;plim\left({\hat{\beta }}_{AS_{n}}\right)=\beta +plim\left\{n^{-1}\;{\left[(X^{\prime }V-A_{AS})X\right]}^{-1}\right\}plim\left\{n^{-1}(X^{\prime }V-A_{AS})\left[f+\varepsilon \right]\right\}.$$

Because f can be counted as a nuisance parameter, and from assumptions (i) and (ii), $plim\left\{n^{-1}\;{\left[(X^{\prime }V-A_{AS})X\right]}^{-1}\right\}=F_{n}^{-1}$ and $plim\left\{n^{-1}(X^{\prime }V-A_{AS})\left[f+\varepsilon \right]\right\}=o\left(1\right)$. Therefore, the expression at the right side in (D1) goes to zero. Thus, from that, it is obtained that:

(A10) $$\mathrm{argmin}\left(\psi_{n}\right)\overset{p}{\to }\mathrm{argmin}\left(\psi \right),\;\;\;\;{\hat{\beta }}_{AS_{n}}\overset{d}{\to }\beta .$$

Note that the results obtained above are for τ≥1, which means $\psi_{n}$ has a convex structure (see [34,35]). However, the proposed AS estimator includes the case of τ<1, so that $\psi_{n}$ is not convex. In this matter, Equation (D2) is processed differently as:

(A11) $$\psi_{n}\left({\hat{\beta }}_{AS_{n}},\hat{f}\left(s_{t}\right)\right)>n^{-1}\overset{n}{\underset{t=1}{\sum }}{\left[Y_{t}-X_{t}{\hat{\beta }}_{AS_{n}}-\hat{f}\left(s_{t}\right)\right]}^{2}=\;\psi_{n}^{\left(0\right)}\left({\hat{\beta }}_{AS_{n}},\hat{f}\left(s_{t}\right)\right)$$

Note that Equation (D3) is validated for all ${\hat{\beta }}_{AS_{n}}$. Moreover, $argmin\left(\psi_{n}\right)=O_{p}\left(1\right)$, because $\left(\psi_{n}^{\left(0\right)}\right)=O_{p}\left(1\right)$. □

## Appendix E

**Proof** **of** **Theorem** **3.** To show the proof of Theorem 3, due to the non-convex structure of τ<1, some complex expressions are needed for minimization criterion ξ. These are given by:

(A12) $$\xi_{n}\left(\theta \right)=\sum_{t=1}^{n}\left[{\left(\varepsilon_{t}-\frac{\theta^{T}X_{t}}{n^{-1}}\right)}^{2}-\varepsilon_{t}\right]+\lambda_{n}\sum_{j=1}^{p}\left[{\left|\beta_{j}+\frac{\theta_{j}}{n^{-1}}\right|}^{\tau }-{\left|\beta_{j}\right|}^{\tau }\right].$$

Due to $\lambda_{n}=O\left(n^{\tau /2}\right)=o\left(\sqrt{n}\right)$, the following expression is obtained similar to Theorem 3:

(A13) $$\lambda_{n}\sum_{j=1}^{p}\left[{\left|\beta_{j}+\frac{\theta_{j}}{n^{-1}}\right|}^{\tau }-{\left|\beta_{j}\right|}^{\tau }\right]\overset{d}{\to }\lambda_{0}\sum_{j=1}^{p}{\left|\theta_{j}\right|}^{\tau }I\left(\beta_{j}=0\right).$$

Then the convergence is realized as follows:

(A14) $$argmin\left(\xi_{n}\right)\overset{d}{\to }argmin\left(\xi \right).$$

Thus, the proof is finished. It is important to note that, for τ<1, the non-zero regression coefficients of the model can be estimated without asymptotic bias if zero ones are shrunk to the zero with a positive probability. □
